# UV irradiation induces homologous recombination genes in the model archaeon, *Halobacterium *sp. NRC-1

**DOI:** 10.1186/1746-1448-1-3

**Published:** 2005-07-04

**Authors:** Shirley McCready, Jochen A Müller, Ivan Boubriak, Brian R Berquist, Wooi Loon Ng, Shiladitya DasSarma

**Affiliations:** 1 School of Biological Molecular Sciences, Oxford Brookes University, Oxford OX3 0BP, UK; 2 Center of Marine Biotechnology, University of Maryland Biotechnology Institute, 701 E. Pratt St., Suite 236, Baltimore, MD 21202 USA; 3 Molecular and Structural Biology Program, Greenebaum Cancer Center, University of Maryland, Baltimore, MD 21201, USA

## Abstract

**Background:**

A variety of strategies for survival of UV irradiation are used by cells, ranging from repair of UV-damaged DNA, cell cycle arrest, tolerance of unrepaired UV photoproducts, and shielding from UV light. Some of these responses involve UV-inducible genes, including the SOS response in bacteria and an array of genes in eukaryotes. To address the mechanisms used in the third branch of life, we have studied the model archaeon, *Halobacterium *sp. strain NRC-1, which tolerates high levels of solar radiation in its natural hypersaline environment.

**Results:**

Cells were irradiated with 30–70 J/m^2 ^UV-C and an immunoassay showed that the resulting DNA damage was largely repaired within 3 hours in the dark. Under such conditions, transcriptional profiling showed the most strongly up-regulated gene was *radA1*, the archaeal homolog of *rad51*/*recA*, which was induced 7-fold. Additional genes involved in homologous recombination, such as *arj1 *(*recJ*-like exonuclease), *dbp *(eukaryote-like DNA binding protein of the superfamily I DNA and RNA helicases), and *rfa3 *(replication protein A complex), as well as *nrdJ*, encoding for cobalamin-dependent ribonucleotide reductase involved in DNA metabolism, were also significantly induced in one or more of our experimental conditions. Neither prokaryotic nor eukaryotic excision repair gene homologs were induced and there was no evidence of an SOS-like response.

**Conclusion:**

These results show that homologous recombination plays an important role in the cellular response of *Halobacterium *sp. NRC-1 to UV damage. Homologous recombination may permit rescue of stalled replication forks, and/or facilitate recombinational repair. In either case, this provides a mechanism for the observed high-frequency recombination among natural populations of halophilic archaea.

## Background

In all organisms studied to date, UV irradiation causes inducible responses. In some bacteria the inducible SOS response involves about 40 genes that are up-regulated dependent on the pleiotropic regulator, LexA [1]. In eukaryotes a variety of genes are up- and down-regulated in response to UV-damage including several DNA repair genes, although no eukaryotic equivalent of the bacterial SOS response has been identified [2]. Among archaea, most studies have heretofore been limited to comparative genomic approaches where inducible mechanisms have not been discernable [3,4]. The hyperthermophilic archaea have been found to carry homologs of several eukaryotic nucleotide excision repair (NER) genes, including *rad2 *(*FEN1*/*XPG*), *rad3 *(*XPD*), *eif4A *(*rad1*/*XPF*), and *rad25 *(*XPB*) [5,6]. Remarkably, extremely halophilic archaea, such as the model organism *Halobacterium *sp. strain NRC-1, and some non-thermophilic methanogenic archaea, were found to harbor homologs of both eukaryotic NER genes and bacterial NER genes, *uvrA*, *uvrB*, *uvrC *and *uvrD *[6-8]. The classic SOS response system regulated by LexA is lacking in archaea, however, and the relationship between the bacterial and eukaryotic repair systems in archaea is not currently known [9].

**Figure 1:**
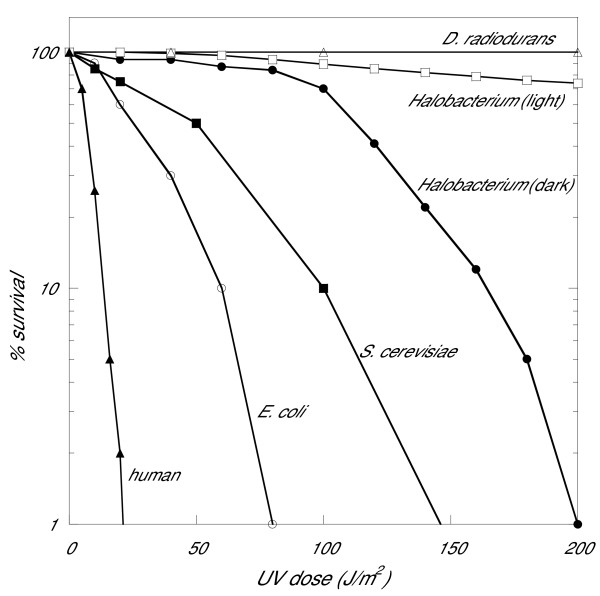
Survival of model organisms exposed to UV-C radiation. The percent survival (*y *axis logarithmic scale) is plotted versus dose of UV radiation (*x *axis linear scale) for human fibroblasts [41], *Escherichia coli *[42], *Saccharomyces cerevisiae *[43], *Halobacterium *sp. NRC-1 (in the dark or in presence of visible light) [11], and *Deinococcus radiodurans *[44].

Halophilic archaea, such as *Halobacterium *spp., are excellent experimental systems for studies of DNA repair because they are amongst the few archaeal microorganisms to encounter high levels of sunlight in their natural environment. They occupy an extreme environmental niche, where exposure to intense solar radiation leads to evaporation and concentration of NaCl to near- or even super-saturation. These microorganisms, including the sequenced wild-type model, *Halobacterium *sp. NRC-1, are highly resistant to the damaging effects of UV radiation in sunlight, principally due to extremely efficient photoreactivation of DNA damage [10,11]. In the presence of visible light they can survive UV doses many times higher than they would ever be exposed to naturally. Cell survival approaches 100 % after doses up to 100 J/m^2 ^while 1 hour exposure to sunlight inflicts damage equivalent to, at most, only a few J/m^2 ^[[12]; SM, unpublished]. UV tolerance of *Halobacterium *sp. NRC-1 is compared to other key organisms in Figure 1. *Halobacterium *is significantly more UV-tolerant, even without photoreactivating light, than *Escherichia coli *or *Saccharomyces cerevisiae*, though not as resistant as the extremely radiation-resistant *Deinococcus radiodurans*. Halophilic archaea also have excision repair mechanisms that can operate in the absence of photoreactivating light [12,13]. In addition, like bacteria and eukaryotes, they are likely to possess mechanisms that enable them to tolerate the presence of some unrepaired UV lesions, including lesion bypass by DNA polymerases that can circumvent photoproducts [14]) and recombination to facilitate recovery of stalled replication forks [15,16].

We have taken a multifaceted approach to the study of UV responses in *Halobacterium *sp. NRC-1. Previously, the critical role for *phr2 *in light repair was demonstrated through a combination of genetics and biochemistry [11]. Here, we use DNA microarrays to show the importance of homologous recombination genes in the response of cells to UV damage. Interestingly, our results are distinct from those obtained in a previous study on *Halobacterium *sp. NRC-1 using significantly higher doses of UV irradiation [17].

## Results and Discussion

**Figure 2:**
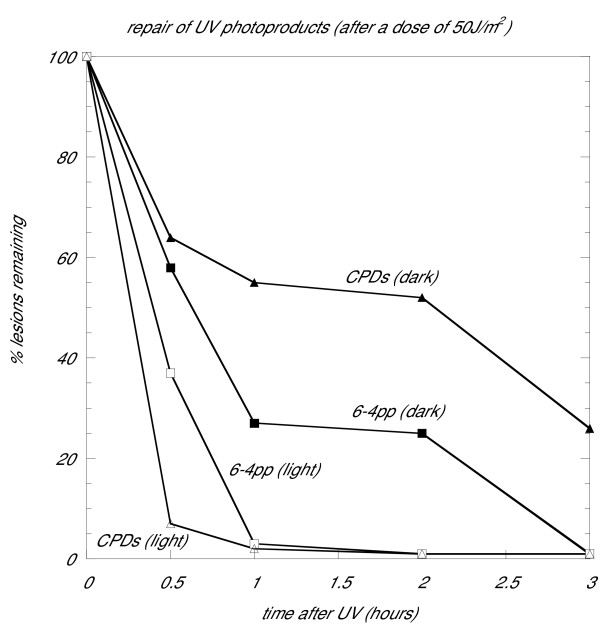
Repair of the two principle UV-induced photoproducts in *Halobacterium *sp. Repair of cyclobutane pyrimidine dimers (CPDs) and 6-4 photoproducts (6-4 pp) was measured after a UV-C dose of 50 J/m^2^. About 55 % of CPDs and 25 % of 6-4 pp remain unrepaired after 1 hour; after 3 hours, the percentages are 28% and 2% respectively.

In order to understand gene expression responses to UV at the whole genome level, we studied the model archaeon, *Halobacterium *sp. strain NRC-1, employing DNA microarrays. We used three different doses of UV-C, 30 J/m^2^, 50 J/m^2^, and 70 J/m^2^, with post-irradiation incubation times in the dark of 1 hour and 3 hours (Materials and Methods). At these UV doses, survival of cells is close to 100 % following DNA damage either in the light or in the dark (Figure 1) [11,18]. Cells were irradiated in growth medium with post-irradiation incubation in the same medium so as to introduce minimal additional stresses. To give an indication of repair rates at the doses used, we determined the relative occurrences of cyclobutane pyrimidine dimers (CPDs) and 6-4 photoproducts over time after irradiation with an intermediate dose of 50 J/m^2 ^(Figure 2). The majority of photoproducts were repaired during the 3-hour period, though some cyclobutane dimers still remained at 3 hours.

Custom DNA microarrays were fabricated using inkjet technology (Agilent Technologies, Palo Alto, CA) with in-house oligonucleotide design performed with the program OligoPicker [19]. The arrays contained 8,455 60-mer nucleotide features representing 2474 open reading frames (ORFs). The high specificity of 60-mer oligonucoletide arrays have been demonstrated previously [20]. Up to three probes were designed per ORF with a mean T_*m *_of 81°C and a T_*m *_range of 3°C. The microarray slides harbor both gene-probes (~8,000 features per array) as well as ~400 negative and positive control spots to test hybridization conditions and allow for error modeling. These microarrays were thoroughly tested for linearity of response and statistical significance in a related study of anaerobic respiration in *Halobacterium *sp. NRC-1 [21]. Signal intensities with a dynamic range in excess of three orders of magnitude were found allowing simultaneous analysis of low- and high intensity features.

For microarray analysis, two doses of UV-C, 30 J/m^2 ^and 70 J/m^2 ^were used. Microarray data quality was considered high. After removal of outliers, features replicated within a single array showed low differences in absolute processed signal intensities (7 % on average); and spot-to-spot variation for replicate experiments was 9 %. Of the 2474 open reading frames (ORFs) of *Halobacterium *sp. NRC-1 represented, 100 were significantly up-regulated and 150 were significantly down-regulated (1.5-fold above or below, respectively, p-value < 0.05) in at least one experimental setting. In the current study, we focused on genes involved in homologous recombination and DNA metabolism, which are the most significantly induced (Table 1). Expression levels for all genes represented on the array for the 30 J/m^2 ^dose are also provided (see Additional file 1). Data obtained from UV irradiation with 70 J/m^2 ^were essentially the same (not shown). At either dose, the pattern of inducible transcripts was very different from what would be seen in *E. coli*, not surprisingly, since there is no LexA homolog in *Halobacterium *sp. NRC-1. Moreover, there was no evidence of a classic coordinated SOS response and neither the prokaryotic DNA repair genes, including *uvrA*, *uvrB*, *uvrC *and *uvrD *nor any of the eukaryotic repair gene homologs were up-regulated.

**Table 1:** UV irradiation (30 J/m^2^)-inducible recombination- and DNA metabolism genes in *Halobacterium *sp. NRC-1.

Functional category	Gene number	Gene name	Fold Induction		Predicted function
			1 hr	3 hr	
recombination	VNG2473	*radA1*	7.4	6.4	Rad51/RecA recombinase
	VNG2160	*rfa3*	1.5^*a*^	1.5^*a*^	RPA41 homolog, contains Zn- finger motif
	VNG2162	*rfa8*	1.5^*a*^	1.5^*a*^	RPA32 homolog
	VNG2163	*ral*	1.5^*a*^	1.5^*a*^	RPA linked ORF
	VNG2167	*dbp*	1.9	1.4	Superfamily I helicase, DNA binding protein eukaryotic-like
	VNG0779	*arj1*	1.4	1.5	RecJ like exonuclease
					
DNA	VNG1644	*nrdJ*	2.8	3.1	class II ribonucleoside reductase
metabolism	VNG1642	Vng1642	3.5	4.3	Unknown, contains Zn- finger motif

^*a *^Fold induction after irradiation with 70 J/m^2^.

**Figure 3:**
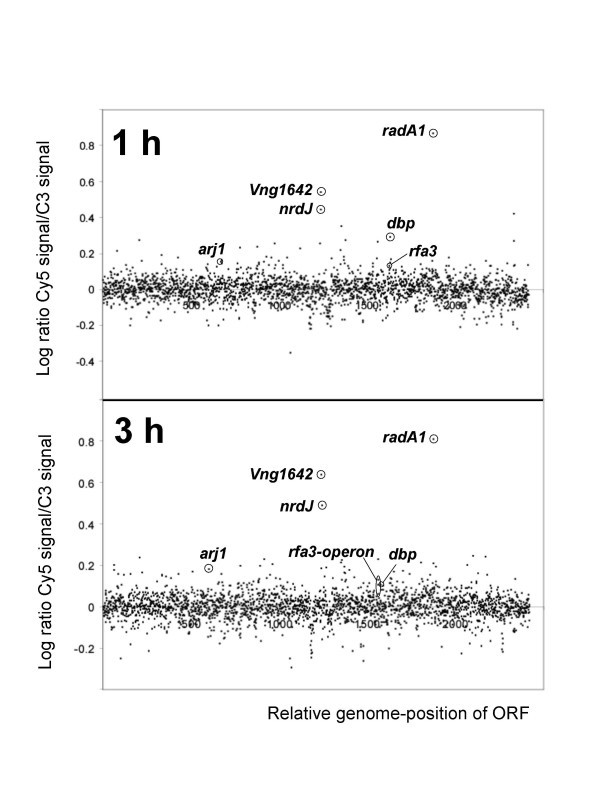
Whole genome microarray hybridization results comparing UV-irradiated cells to control cells. Irradiated cells received a UV-C dose of 30 J/m^2 ^and incubated in the dark for 1 h (upper panel) and 3 h (lower panel). Control cells were treated exactly the same except for UV-irradiation. For each ORF represented on the array, the logarithm of the hybridization ratio of UV-irradiated cells (Cy5-labeled cDNA) to control cells (Cy3-labeled cDNA) is displayed in black marks on the *y *axis. The location of ORFs within the entire 2.6-megabase genome maps on the *x *axis. Expression ratios of selected genes are indicated.

Strikingly, however, the *radA1 *gene showed the most inducible transcript (~7 fold) at 1 hr and 3 hrs after both UV doses (Table 1; Figure 3). RadA1 is the *Halobacterium *sp. NRC-1 homolog of RecA/Rad51, which catalyses strand invasion and exchange during homologous recombination. RadA1 is more similar to Rad51 in eukaryotes than to bacterial RecA. Deletion of *radA *has been shown to cause severe UV sensitivity in the related halophililc archaeon, *Haloferax volcanii *[22]. The expression change of *radA1 *was considered statistically highly significant based on the following: The *radA1 *gene is represented by 3 different probes per array with one of those probes being duplicated, combining to a total of 24 data points for that gene in the present report. In addition, cDNA was prepared from three independently treated cultures per condition and array. The average standard deviation of the fold changes for all *radA1*-probes within an array was 0.85, while the array-to-array difference for identical probes in replicated experiments was 18 %. The average p-value of log ratios was < 10^-22^. Results from previous transcriptome profiling after environmental perturbations indicate that a 7-fold expression change is high in comparison to any gene in *Halobacterium *sp. NRC-1 [[21]; JM and SD, unpublished data]. In those experiments (5 different conditions not involving UV irradiation) the *radA1 *gene was not differentially expressed demonstrating that the induction presented here was caused by UV irradiation.

The *radA2 *(*radB*) gene, a second homolog of *recA *in archaea, encoding a protein with unknown role in homologous recombination, was not up-regulated, in agreement with observations in other archaea [23]. Accompanying *radA1 *induction, several other genes involved in homologous recombination were significantly induced after UV irradiation. The *dbp *gene, encoding a eukaryote-like DNA binding protein of the superfamily I DNA and RNA helicases, was upregulated 1.95 (p-value 0.04) at 1 hr post UV-irradiation with 30 J/m^2^. The *arj1 *gene, encoding a *recJ*-like exonuclease was up-regulated 1.5 fold (p-value 0.008) at 3 hrs (30 J/m^2^). Additionally, an apparent operon encoding RPA ssDNA binding protein complex, *rfa3*, (VNG2160, RPA41 homolog, Zn-finger containing), *rfa8 *(VNG2162, RPA32 homolog), and an uncharacterized linked ORF (VNG2163) was induced 1.53 ± 0.02 fold (p-values < 0.001) at both time points after irradiation with 70 J/m^2^. Similar fold changes were measured after irradiation with 30 J/m^2^, however, p-values were around 0.1. In eukaryotes, RPA-ssDNA complexes are formed during almost all DNA-damage repair pathways. For example, RPA-proteins are recruited to Rad51 foci, protein complexes that accumulate at sites of DNA damage and stalled replication forks [24,25]. None of the other four RPA homologs of *Halobacterium *sp. NRC-1 (VNG0133, 1253, 1255, 6403) was significantly differentially expressed. As for *radA1*, neither of the above genes were found to be differentially expressed in other environmental perturbation experiments [[21]; JM and SD, unpublished data].

Our microarray data, together with these findings, strongly suggest a key role for homologous recombination in survival of UV damage in this class of archaea. By analogy with other organisms, there are at least two ways that recombination might contribute to survival of UV damage in *Halobacterium *sp. NRC-1. (i) Recombinational rescue of stalled replication forks may take place [15,26]. Rescue of stalled forks is likely to be error-free and this would fit with the low mutation rate in *Halobacterium *spp. [[1]27; SM, unpublished data]. (ii) Recombinational repair may occur by using duplicate copies of the genome, a mechanism that has been suggested to facilitate post-irradiation survival of *D. radiodurans *[28]. *Halobacterium *sp. NRC-1 is thought to contain multiple copies of its genome (J. Soppa pers. com.), which may greatly enhance recombinational repair.

In addition to genes involved in homologous recombination, several other genes were also highly induced. Most interestingly, the gene encoding a cobalamin-dependent class II RNR (*nrdJ*, formerly *nrdB2*) was strongly up-regulated (Figure 3, Table 1). A small open reading frame (VNG1642) located immediately downstream to *nrdJ *encoding a small uncharacterized zinc-finger containing protein (COG1645) was also strongly induced. RNR is the rate-limiting enzyme in the *de novo *synthesis of deoxyribonucleotide triphosphates which are utilized in both DNA synthesis and DNA repair; the up-regulation of the *nrd *genes may reflect the need for increased deoxynucleotide concentrations to allow rapid and accurate excision repair. In yeast, DNA damage elicits an increase in dNTP levels; and increased dNTP pools improve cell survival post DNA damage [29]. In many other organisms, RNR genes are up-regulated by UV and were among the very first UV-inducible genes identified in an early study of UV-inducible promoters in budding yeast [30]. RNR is regulated during the cell cycle in eukaryotes and the UV response depends on binding of transcription factor E2F to sites in the promoter [31]. It is noteworthy that, in *E. coli*, *nrd *genes are UV-inducible even in the absence of *lexA *[1].

In addition to *nrdJ*, *arcBCA*, required for fermentation of arginine [32] were also particularly strongly induced, though only at the early time, and with some variation in magnitude of induction between repeat experiments. Whether this induction reflects a demand for rapid supply of ATP during periods of DNA-damage repair is not known. Another possibility is that the expression of these genes is exquisitely responsive to small stresses and we view these results with some caution at this stage.

A previous transcriptome analysis of UV-irradiated *Halobacterium *sp. strain NRC-1 cultures [17] showed, as we do, the lack of an SOS-like response and the lack of up-regulation of any of the NER genes by UV. However, these authors did not report strong induction of recombination genes or of RNR genes. The UV dose used in the latter study was substantially higher (200 J/m^2^). This dose, which induces, approximately one photoproduct per 600 bp of DNA, causes about one hundred times as much DNA damage as induced by natural sunlight and resulted in compromised cell survival. It may be that the high UV dose caused interference with gene expression. This is in contrast to approximately one photoproduct per 4 kb at 30 J/m^2 ^and one per 1.7 kb at 70 J/m^2 ^[33-35], doses tolerated by *Halobacterium *sp. NRC-1 and used in the current study.

There have been questions raised as to the significance of gene expression responses to UV and other DNA damaging agents. In the yeast, *S. cerevisiae*, an analysis of deletion mutants lacking UV-inducible genes suggested that most do not contribute to survival of UV irradiation [36]. In addition, most yeast genes identified as having a role in surviving UV damage, by isolation of UV-sensitive mutants, including most NER genes, are not UV-inducible. In human cells too, most repair genes are not UV-inducible [37]. Thus, transcriptome analysis must be interpreted with caution and inferences about the extent of gene involvement should be supported by physiological and functional studies. In the present report, the absolute contribution of the different DNA-damage repair systems has not been investigated. However, the high level of up-regulation of *radA1 *and induction of other recombination genes while comparable few other gene expression changes occur, clearly suggests a significant role of homologous recombination in DNA-damage repair in *Halobacterium *sp. NRC-1.

## Conclusion

Our transcriptome profiling work, together with our studies of the physiological response of a model archaeon, has shown that genes involved in homologous recombination are induced by UV irradiation in relatively low doses. Our results are consistent with homologous recombination playing an important role in the cellular response to UV damage in *Halobacterium *sp. NRC-1, either to permit rescue of stalled replication forks or to facilitate recombinational repair. In either case, we find that induction of recombination genes is prominent in the response of *Halobacterium *sp. NRC-1 to UV irradiation, which is particularly significant as it has been shown recently that recombination is important in facilitating genetic exchange in wild populations of halophilic archaea [38]. Our results suggest that homologous recombination is stimulated by sunlight in this model archaeon.

## Methods

### Culture conditions and UV-irradiation

*Halobacterium *sp. strain NRC- 1 was grown at 37°C under aerobic conditions to early exponential growth phase (OD_600 _0.19 – 0.23) in complete medium [39] and irradiated in the dark, in medium, using a UV-C source with dose rate of 1 J/sec/m^2^. Post-UV incubation was continued at the same temperature, without changing the medium. Typically, UV-irradiation is not carried out in growth media because of possible absorption of UV wavelengths. For the present microarray experiments, irradiation in growth medium was of particular importance to avoid additional stress caused to the cells by harvesting and changing media. Moreover, we ascertained that the absorption of UV-C by the growth medium used was minimal by measuring transmission of 260 nm light in a spectrophotometer. The effective UV dose was not significantly affected by irradiating in medium.

### Measurement of photoproducts

A dot-blot immunoassay for differential quantitation of CPDs and 6-4 photoproducts was carried out as described in detail previously [40]. Briefly, exponential phase cells were harvested and irradiated in sterile salts solution with UV-C at a dose rate of 1 J/sec m^2^. Aliquots of yeast extract and casein hydrolysate solutions were added to restore nutrients and the irradiated cells were incubated aerobically at 37°C to allow repair to proceed. All irradiation and post-UV incubation was carried out either under yellow light illumination or in the dark. Samples were taken at timed intervals and DNA extracted for measurement of photoproducts. DNA concentrations in different samples were carefully equalised. Subsequently, each DNA sample was divided into two and one half was treated with hot NaOH to destroy 6-4 photoproducts. Two identical dot blots were prepared on nitrocellulose filters, each containing a set of dilutions of each DNA sample. One filter was exposed to a CPD photolyase to destroy cyclobutane dimers in all the DNA samples on that blot to allow measurement of 6-4 photoproducts alone. The dot blots were then exposed to rabbit polyclonal antiserum containing antibodies to 6-4 photoproducts and CPDs, then to biotinylated anti-rabbit antibody followed colorimetric quantitation using alkaline photsphatase-conjugated Extravidin (Sigma), Nitro Blue tetrazolium (NBT) and 5-bromo-4-chloro-indolyl phosphate (BCIP) substrate. The amount of color was measured using a scanning densitometer (BioRad GS-670) and compared to a set of standards included on each blot.

### Microarray procedures

Relative mRNA levels were determined by parallel two-color hybridization to oligonucleotide (60-mer) microarrays representing 2474 ORFs representing 92 % of *Halobacterium *sp. NRC-1 ORFs according to Müller and DasSarma [21]. Transcriptome profiling of cells irradiated with 30 J/m^2 ^was carried out in duplicate. Transcriptome analysis of cells irradiated with 70 J/m^2 ^was carried out only once since results were essentially the same as for 30 J/m^2^. Total RNA was isolated from 50 ml cultures immediately after harvesting at 2°C using Agilent Total RNA isolation kit (Agilent) and DNA was hydrolysed using amplification grade DNase (Sigma, UK). In order to minimize biological noise, RNA preparations from three cultures grown under identical conditions were pooled to equal parts for cDNA synthesis. cDNA was prepared from 7 μg total RNA with Super Script III reverse transcriptase (Invitrogen, UK) and Cy3- or Cy5-dCTP (Amersham Biosciences, UK). Performance of duplicate experiments in which dyes were swapped during synthesis to account for labelling differences was not required. Previous results showed that differences in the relative intensity of the channels could be adjusted for by intensity-dependent LOWESS normalization [[21]; JM and SD, unpublished data]. cDNA preparations were purified after alkaline hydrolysis of RNA on Qiagen mini-elute columns (Qiagen, UK). The labeled cDNA targets were mixed with hybridization buffer (Agilent) and control targets (Agilent), and hybridized to microarray slides, assembled into a hybridization chamber (Agilent), for 17 h at 60°C in the dark. Post hybridization, the slides were washed as described [21] and scanned for the Cy3 and Cy5 fluorescent signals with an Agilent DNA-microarray scanner (Model no. G2565BA). Image processing and statistical analysis were carried out using Agilent Feature Extraction Software Version 7.1 as described previously [21]. Log ratios for each feature were calculated and the significance of the log ratio was assessed by calculating the most conservative log ratio error and significance value (p-value) using a standard error propagation algorithm (Agilent) and a universal error model (Rosetta Biosoftware).

## Competing interests

The authors declare that they have no competing interests.

## Authors' contributions

SM designed the experiments, analyzed the data, and drafted the manuscript. JM assisted with experimental design, conducted the DNA microarray hybridization, and carried out bioinformatics and data analysis. IB assisted with experimental design and conducted the UV irradiation, RNA preparation, and cDNA labelling. BB assisted with bioinformatics analysis. WN assisted with UV irradiation, RNA preparation, and cDNA labelling. SD designed the experiments, assisted with data interpretation, and prepared the manuscript.

## Supplementary Material

Additional File 1 Expression changes of *Halobacterium *NRC-1 open reading frames after UV irradiation with 30 J/m^2 ^is provided as an additional XLS file. Click here for file

## References

[B1] Courcelle J Khodursky A Peter B Brown PO Hanawalt PC Comparative gene expression profiles following UV exposure in wild-type and SOS-deficient *Escherichia coli* Genetics 2001 158 1 41 64 11333217 10.1093/genetics/158.1.41 PMC1461638

[B2] Cline SD Hanawalt PC Who's on first in the cellular response to DNA damage? Nat Rev Mol Cell Biol 2003 4 5 361 372 10.1038/nrm1101 12728270

[B3] Eisen JA Hanawalt PC A phylogenomic study of DNA repair genes, proteins, and processes Mutat Res 1999 435 3 171 213 10606811 10.1016/s0921-8777(99)00050-6 PMC3158673

[B4] DasSarma S Kennedy SP Berquist B Ng WV Baliga NS Spudich JL Krebs MP Eisen JA Johnson CH Hood L Genomic perspective on the photobiology of *Halobacterium *species NRC-1, a phototrophic, phototactic, and UV-tolerant haloarchaeon Photosyn Res 2001 70 3 17 10.1023/A:1013879706863 16228359

[B5] Grogan DW The question of DNA repair in hyperthermophilic archaea Trends Microbiol 2000 8 4 180 185 10.1016/S0966-842X(00)01729-7 10754577

[B6] Grogan DW Stability and repair of DNA in hyperthermophilic archaea Curr Issues Mol Biol 2004 6 2 137 144 15119824

[B7] DasSarma S Genome sequence of an extremely halophilic archaeon Microbial Genomes 2004 383 399

[B8] Ng V Genome sequence of *Halobacterium * species NRC-1 Proc Natl Acad Sci U S A 2000 97 22 12176 12181 10.1073/pnas.190337797 11016950 PMC17314

[B9] Walker GC Understanding the complexity of an organism's responses to DNA damage Cold Spring Harb Symp Quant Biol 2000 65 1 10 10.1101/sqb.2000.65.1 12760015

[B10] Hescox MA Carlberg DM Photoreactivation in *Halobacterium cutirubrum* Can J Microbiol 1972 18 7 981 985 5070716 10.1139/m72-152

[B11] McCready SJ Marcello L Repair of UV damage in *Halobacterium salinarum* Bioch Soc Trans 2003 31 3 694 698 10.1042/BST0310694 12773185

[B12] Quaite FE Takayanagi S Ruffini J Sutherland JC Sutherland BM DNA damage levels determine cyclobutyl pyrimidine dimer repair mechanisms in Alfalfa seedlings Plant Cell 1994 6 11 1635 1641 10.1105/tpc.6.11.1635 12244228 PMC160549

[B13] McCready SJ The repair of ultraviolet light-induced DNA damage in the halophilic archaebacteria, *Halobacterium cutirubrum*, *Halobacterium halobium *and *Haloferax volcanii* Mutat Res 1996 364 1 25 32 8814335 10.1016/0921-8777(96)00018-3

[B14] Boudsocq F Iwai S Hanaoka F Woodgate R *Sulfolobus solfataricus *P2 DNA polymerase IV (Dpo4): an archaeal DinB-like DNA polymerase with lesion-bypass properties akin to eukaryotic polη Nucleic Acids Res 2001 29 22 4607 4616 10.1093/nar/29.22.4607 11713310 PMC92520

[B15] Kuzminov A DNA replication meets genetic exchange: chromosomal damage and its repair by homologous recombination Proc Natl Acad Sci U S A 2001 98 15 8461 8468 10.1073/pnas.151260698 11459990 PMC37458

[B16] Courcelle J Donaldson JR Chow KH Courcelle CT DNA damage-induced replication fork regression and processing in *Escherichia coli* Science 2003 299 5609 1064 1067 10.1126/science.1081328 12543983

[B17] Baliga NS Bjork SJ Bonneau R Pan M Iloanusi C Kottemann MC Hood L DiRuggiero J Systems level insights into the stress response to UV radiation in the halophilic archaeon *Halobacterium *NRC-1 Genome Res 2004 14 6 1025 1035 10.1101/gr.1993504 15140832 PMC419780

[B18] Fitt PS Sharma N Castellanos G A comparison of liquid-holding recovery and photoreactivation in halophilic and non-halophilic bacteria Biochim Biophys Acta 1983 739 1 73 78 6338926 10.1016/0167-4781(83)90046-5

[B19] Wang X Seed B Selection of oligonucleotide probes for protein coding sequences Bioinformatics 2003 19 7 796 802 10.1093/bioinformatics/btg086 12724288

[B20] Hughes TR Expression profiling using microarrays fabricated by an ink-jet oligonucleotide synthesizer Nature Biotechnol 2001 19 4 342 347 10.1038/86730 11283592

[B21] Müller JA DasSarma S Genomic analysis of anaerobic respiration in the archaeon *Halobacterium *species NRC-1: dimethyl sulfoxide and trimethylamine *N*-oxide as terminal electron acceptors J Bacteriol 2005 187 5 1659 1667 10.1128/JB.187.5.1659-1667.2005 15716436 PMC1064022

[B22] Woods WG Dyall-Smith ML Construction and analysis of a recombination-deficient (*radA*) mutant of *Haloferax volcanii* Mol Microbiol 1997 23 4 791 797 10.1046/j.1365-2958.1997.2651626.x 9157249

[B23] Reich CI McNeil LK Brace JL Brucker JK Olsen GJ Archaeal RecA homologues: different response to DNA-damaging agents in mesophilic and thermophilic archaea Extremophiles 2001 5 4 265 275 10.1007/s007920100197 11523896

[B24] Golub EI Gupta RC Haaf T Wold MS Radding CM Interaction of human rad51 recombination protein with single-stranded DNA binding protein, RPA Nucleic Acids Res 1998 26 23 5388 5393 10.1093/nar/26.23.5388 9826763 PMC148005

[B25] Tashiro S Walter J Shinohara A Kamada N Cremer T Rad51 accumulation at sites of DNA damage and in postreplicative chromatin J Cell Biol 2000 150 2 283 291 10.1083/jcb.150.2.283 10908572 PMC2180223

[B26] Cox MM Historical overview: searching for replication help in all of the rec places Proc Natl Acad Sci U S A 2001 98 15 8173 8180 10.1073/pnas.131004998 11459950 PMC37418

[B27] Martin EL Reinhardt RL Baum LL Becker MR Shaffer JJ Kokjohn TA The effects of ultraviolet radiation on the moderate halophile *Halomonas elongata *and the extreme halophile *Halobacterium salinarum* Can J Microbiol 2000 46 2 180 187 10.1139/cjm-46-2-180 10721487

[B28] Makarova KS Aravind L Wolf YI Tatusov RL Minton KW Koonin EV Daly MJ Genome of the extremely radiation-resistant bacterium *Deinococcus radiodurans *viewed from the perspective of comparative genomics Microbiol Mol Biol Rev 2001 65 1 44 79 10.1128/MMBR.65.1.44-79.2001 11238985 PMC99018

[B29] Chabes A Georgieva B Domkin V Zhao X Rothstein R Thelander L Survival of DNA damage in yeast directly depends on increased dNTP levels allowed by relaxed feedback inhibition of ribonucleotide reductase Cell 2003 112 3 391 401 10.1016/S0092-8674(03)00075-8 12581528

[B30] Ruby SW Szostak JW Specific *Saccharomyces cerevisiae *genes are expressed in response to DNA-damaging agents Mol Cell Biol 1985 5 1 75 84 3920512 10.1128/mcb.5.1.75-84.1985 PMC366680

[B31] Lincker F Philipps G Chaboute ME UV-C response of the ribonucleotide reductase large subunit involves both E2F-mediated gene transcriptional regulation and protein subcellular relocalization in tobacco cells Nucleic Acid Res 2004 32 4 1430 1438 10.1093/nar/gkh310 14990748 PMC390297

[B32] Ruepp A Soppa J Fermentative arginine degradation in *Halobacterium salinarium *(formerly *Halobacterium halobium*): genes, gene products, and transcripts of the *arcRACB *gene cluster J Bacteriol 1996 178 16 4942 4947 8759859 10.1128/jb.178.16.4942-4947.1996 PMC178278

[B33] Unrau P Wheatcroft R Cox B Olive T The formation of pyrimidine dimers in the DNA of fungi and bacteria Biochim Biophys Acta 1973 312 4 626 632 4200353 10.1016/0005-2787(73)90065-8

[B34] Williams JI Cleaver JE Excision repair of ultraviolet damage in mammalian cells. Evidence for two steps in the excision of pyrimidine dimers Biophys J 1978 22 2 265 279 656544 10.1016/S0006-3495(78)85488-5 PMC1473437

[B35] McCready SJ Cox BS Repair of 2 μm plasmid DNA in *Saccharomyces cerevisiae* Curr Genet 1980 2 3 207 210 10.1007/BF00435687 24189911

[B36] Birrell GW Brown JA Wu HI Giaever G Chu AM Davis RW Brown JM Transcriptional response of *Saccharomyces cerevisiae *to DNA-damaging agents does not identify the genes that protect against these agents Proc Natl Acad Sci U S A 2002 99 13 8778 8783 10.1073/pnas.132275199 12077312 PMC124375

[B37] Rieger KE Chu G Portrait of transcriptional responses to ultraviolet and ionizing radiation in human cells Nucleic Acids Res 2004 32 16 4786 4803 10.1093/nar/gkh783 15356296 PMC519099

[B38] Papke RT Koenig JE Rodriguez-Valera F Doolittle WF Frequent recombination in a saltern population of *Halorubrum* Science 2004 306 5703 1928 1930 10.1126/science.1103289 15591201

[B39] DasSarma S Fleischmann EM Halophiles 1995 Cold Spring Harbor Laboratory Press, Plainview, NY

[B40] McCready SJ Henderson DS A dot blot immunoassay for UV photoproducts Methods in Molecular Biology: DNA Repair Protocols: Eukaryotic Systems 1999 Humana Press 10.1385/1-59259-675-4:147 10443417

[B41] Bohr VA Okumoto DS Hanawalt PC Survival of UV-irradiated mammalian cells correlates with efficient DNA repair in an essential gene Proc Natl Acad Sci U S A 1986 83 11 3830 3833 3459159 10.1073/pnas.83.11.3830 PMC323617

[B42] Schenley RL Fisher WD Swenson PA Pyrimidine dimer excision in surviving and nonsurviving cells of ultraviolet-irradiated cultures of *Escherichia coli* J Bacteriol 1976 126 2 985 989 770461 10.1128/jb.126.2.985-989.1976 PMC233237

[B43] Tuite MF Cox BS Ultraviolet mutagenesis studies of [*psi*], a cytoplasmic determinant of *Saccharomyces cerevisiae* Genetics 1980 95 3 611 630 7002721 10.1093/genetics/95.3.611 PMC1214250

[B44] Earl AM Rankin SK Kim K-P Lamendola ON Battista JR Genetic evidence that the *uvsE *gene product of *Deinococcus radiodurans *R1 is a UV damage endonuclease J Bacteriol 2002 184 4 1003 1009 10.1128/jb.184.4.1003-1009.2002 11807060 PMC134819

